# Triazine-Based Small Molecules: A Potential New Class of Compounds in the Antifungal Toolbox

**DOI:** 10.3390/pathogens12010126

**Published:** 2023-01-12

**Authors:** Karen A. Conrad, Hyunjeong Kim, Mohammad Qasim, Amel Djehal, Aaron D. Hernday, Laurent Désaubry, Jason M. Rauceo

**Affiliations:** 1 Department of Sciences, John Jay College of the City, University of New York, New York, NY 10019, USA; 2 Department of Molecular and Cellular Biology, School of Natural Sciences, University of California, Merced, CA 95343, USA; 3 Higher National School of Biotechnology of Constantine, Constantine 25100, Algeria; 4 Laboratory of Regenerative Nanomedicine, Center of Research and Biomedicine, University of Strasbourg, 67000 Strasbourg, France

**Keywords:** antifungal, *Candida*, triazine, prohibitin, yeast-to-hyphae transition

## Abstract

Invasive fungal infections caused by *Candida* species remain a significant public health problem worldwide. The increasing prevalence of drug-resistant infections and a limited arsenal of antifungal drugs underscore the need for novel interventions. Here, we screened several classes of pharmacologically active compounds against mammalian diseases for antifungal activity. We found that the synthetic triazine-based compound melanogenin (Mel) 56 is fungicidal in *Candida albicans* laboratory and clinical strains with minimal inhibitory concentrations of 8–16 µg/mL. Furthermore, Mel56 has general antifungal activity in several non-*albicans Candida* species and the non-pathogenic yeast *Saccharomyces cerevisiae*. Surprisingly, Mel56 inhibited the yeast-to-hyphae transition at sublethal concentrations, revealing a new role for triazine-based compounds in fungi. In human cancer cell lines, Mel56 targets the inner mitochondrial integral membrane prohibitin proteins, PHB1 and PHB2. However, Mel56 treatment did not impact *C. albicans* mitochondrial activity, and antifungal activity was similar in prohibitin single, double, and triple homozygous mutant strains compared to the wild-type parental strain. These results suggests that Mel56 has a novel mechanism-of-action in *C. albicans*. Therefore, Mel56 is a promising antifungal candidate warranting further analyses.

## 1. Introduction

The increasing prevalence of antifungal drug resistance caused by *Candida* species is a major global health concern. Mortality and morbidity rates persist at alarmingly high levels in immunocompromised patients suffering with invasive *Candida* infections, despite receiving administration of antifungal therapy [1]. While most cases of invasive candidiasis are caused by a small subset of *Candida* species, *Candida albicans* is the most common human fungal pathogen. Moreover, *C. albicans* causes superficial mucosal infections in the oropharyngeal and vaginal tracts, where approximately 75% of women suffer from vulvovaginal candidiasis at least once in life [2].

Treatment for *C. albicans* infections relies on three classes of drugs—azoles, polyenes, and echinocandins—that target the biosynthesis of the fungal membrane or the cell wall [3,4]. However, *C. albicans* exploits several fitness-enhancing interactions to survive in varying microenvironments and counteract the effects of antifungal drugs. For instance, *C. albicans* can exist in an intricate multicellular community known as a biofilm. Within the biofilm, *C. albicans* thrives in both yeast and hyphal forms and promotes drug resistance via decreasing ergosterol synthesis and increasing the expression of multidrug transporter and stress-response genes [5]. Thus, the urgency to develop new antifungal strategies remains a high priority.

Small molecule screening approaches are highly effective in identifying putative compounds exhibiting intrinsic antifungal activity, or in some instances, to treat drug-resistance when used in combination with antifungal drugs [6,7,8]. The focus of this study is to determine the antifungal properties for various natural and synthetic small molecules that were shown to have excellent therapeutic potential against cancer, neurodegenerative, cardiac, and inflammatory diseases, while having low toxicity in primary cell lines [9,10]. Interestingly, these molecules are described as prohibitin ligands because they bind to mammalian prohibitin proteins [10]. Prohibitins are widely conserved integral membrane proteins that localize to various biological membranes and are members of the SPFH (Stomatin, Prohibitin, Flotillin, HflK/HflC) protein superfamily [11]. In mammals, fungi, parasites, and nematodes, SPFH proteins are associated with mitochondrial functions such as respiratory chain complex assembly, mitophagy (the removal of damaged mitochondria), protein translation, virulence, and apoptosis [12,13,14,15,16].

Prohibitins are targeted by several natural and synthetic compounds that display different profiles of pharmacological activities. Key prohibitin ligands include Mel6 which inhibits melanin production (melanogenesis) in melanocytes [17]. Mel9, Mel41, and Mel56 promote melanogenesis and induce apoptosis in melanoma cancer cell lines [17]. FL3, fluorizoline, and capsaicin induce cancer cell death [18,19]. IN44 inhibits osteoclastogenesis [20], and IM108 protects cardiomyocytes against the adverse effects of doxorubicin [21]. Here, we utilized an in vitro cellular approach to evaluate the antifungal properties of various types of prohibitin inhibitors in *C. albicans*, several non-*albicans Candida* species, and *S*. *cerevisiae*. We provide novel cellular data highlighting the potential of the triazine-based compound Mel56 as an alternative antifungal treatment.

## 2. Materials and Methods

### 2.1. Yeast Strains

The yeast strains *C. albicans* SC5314, *C. albicans* 3147, *C. tropicalis* 1909, *C. parapsilosis* CBS604, *C. dubliniensis* CBS7987, *C. glabrata* CBS138, *S. cerevisiae* BY4741 (*MAT***a** *his3Δ1*, *leu2Δ0*, *met15Δ0*, *uraΔ0*), W303-1A (*MAT***a** *leu2-3*, *112 trp1-1*, *can1-100*, *ura3-1*, *ade2-1*, *his3-11*), and W303-1B (*MAT***α** *leu2-3*, *112 trp1-1*, *can1-100*, *ura3-1*, *ade2-1*, *his3-11*) were purchased from ATCC. *C. albicans* yeast strains MC99 and MC102 were provided by Aaron Mitchell (University of Georgia, USA). *S. cerevisiae* strain BY4742 (*MAT***α** *his3Δ1*, *leu2Δ0*, *met15Δ0*, *uraΔ0*) was obtained from William Chirico (SUNY Downstate, USA). *C. albicans* strain SN250 (*his1Δ/his1Δ*, *leu2Δ*::*C. dubliniensis HIS1/leu2Δ*::*C. maltosa LEU2*, *arg4Δ*/*arg4Δ*, *URA3*/*ura3Δ*::*imm434*, *IRO1*/*iro1Δ*::*imm434*) was previously described [22]. *C. albicans* strains DAY185 [23] and DAY286 [24] were previously described.

### 2.2. Construction of Mutant Strains

Homozygous null mutant *C. albicans* strains were constructed via CRISPR-Cas9 genome editing methods [25,26] using strain SN250 as the parental strain. Oligonucleotides and mutant strain genotypes are listed in Table S1. For each gene deletion, the native open reading frame was replaced with an exogenous CRISPR-Cas9 target sequence, or “AddTag”, as previously described [26]. Custom guide-RNA sequences were designed to target Cas9 cutting within the coding sequence of the gene to be deleted (Table S1) and introduced into pADH139 via PCR stitching as previously described [25]. Synthetic double-stranded donor DNA fragments containing AddTag sequences flanked by 50 bp homology arms that match the sequences immediately upstream and downstream of *PHB1* and *PHB2* were generated by primer extension as previously described [26]. For *PHB12* deletion, a PCR assembly protocol was used to generate donor DNA fragments containing the AddTag sequence flanked by ~200 bp homology arms that match the sequences upstream and downstream of *PHB12* as previously described [27]. Donor DNA fragments were transformed into *C. albicans* along with gRNA and Cas9 expression constructs as previously described [25]. Transformants were selected on YPD supplemented with 200 μg/mL nourseothricin, and replacement of the target gene with an AddTag sequence was verified by colony PCR. After verifying the intended genotype, the Cas9 and gRNA expression cassettes were removed, along with the nourseothricin resistance marker, using the LEUpOUT method [25].

### 2.3. Growth and Minimal Inhibitory Concentration (MIC) Broth Dilution Assays

The prohibitin inhibitors FL3, Mel6, Mel9, Mel41, Mel56, IM108, IN44, fluorizoline, and capsaicin were synthesized according to previously described protocols [17,19,20,21,28]. To prepare stock solutions, chemicals were dissolved in DMSO to final concentrations of 5.0–5.35 mg/mL and stored at −20 °C in a desiccator.

A modified version of the high-throughput screening with antifungal susceptibility method [7] was performed to evaluate the antifungal activity of the inhibitors. In the first screen, yeast cells were treated with three different concentrations of the designated inhibitor to facilitate analysis of multiple yeast strains on a single 96-well plate (Corning, USA).

To prepare the starter cultures, yeast colonies were streaked from frozen stocks onto YPD (1% yeast extract, 2% peptone, 2% dextrose, 2% agar) plates and incubated for 1–2 days at 30 °C. A single colony was inoculated into fresh YPD medium and incubated at 30 °C with shaking at 225 rpm for approximately 16–24 h. Cells were counted with a Countess 3 FL automated cell counter (Life Technologies, USA) and diluted approximately 1:100 in fresh RPMI-1640 medium (or YPD for *S. cerevisiae* cells). For each well, approximately 1.0–3.0 × 10^5^ cells/mL were inoculated into RPMI-1640 medium supplemented with the designated inhibitor to a final volume of 200 µL. Control wells containing cells that were grown in DMSO/RPMI-1640 or RPMI-1640 medium alone were prepared to evaluate the effect of DMSO or no ligand on cell growth. Wells containing the compounds and RPMI-1640 medium without yeast cells were prepared to serve as a “blank” control.

*Candida* strains were grown for 24 h at 30 °C with shaking, and OD_600nm_ readings were acquired every 30 min using a Synergy Mx plate reader (Biotek, USA). *S. cerevisiae* samples were grown for 48 h. The following terms were used to assign yeast growth phenotypes following inhibitor treatment: hypersensitive (no yeast growth is observed in the microplate wells after 24 h of growth), sensitive (growth was observed in the microplate well at approximately less than or equal to 50% of the mean OD_600nm_ value for the corresponding value of the untreated control group), and no effect (growth was the same as untreated control samples). The results were confirmed in at least three independent experiments, and each inhibitor concentration was tested in triplicate. Compounds demonstrating antifungal activity (no observed growth) were reevaluated in at least two independent experiments using freshly synthesized inhibitor prior to MIC testing.

To determine the MIC of a candidate inhibitor, broth microdilution growth assays were performed according to the procedures that were described in the EUCAST definitive document Edef 7.1 [29]. To prepare the inoculum, yeast cultures were prepared as described above in the standard liquid microplate assay. Inhibitors were diluted in RPMI-1640 (50 µL ligand in 1230 µL RPMI-1640) to a final concentration of 128 µg/mL.

Serial double dilutions were performed to prepare the microdilution plate. First, inhibitors were serially diluted two-fold in a 100 µL volume of RPMI-1640 with a starting concentration of 128 µg/mL. Next, 100 µL of cell suspension was added to each well to a final concentration of 1.0–3.0 × 10^5^ cells/mL. The final inhibitor concentration ranged from 0.125 to 64 µg/mL, and the final DMSO concentration ranged from 0.0023% to 1.30%. Control groups and incubation conditions were identical to the standard microplate growth experiments. The results were confirmed in at least two independent experiments, and each inhibitor concentration was tested in triplicate. To compare the antifungal activity of candidate prohibitin ligands against common antifungal drugs, MIC values of 1.0–2.5 µg/mL were obtained for the antifungal drug fluconazole (Sigma, USA) in broth assays using *C. albicans* strains SN250 and SC5314.

### 2.4. Yeast Viability Assays

Yeast cells were grown for approximately 16–24 h at 30 °C with shaking at 225 rpm in YPD. The cells were counted, and approximately 1–3 × 10^7^ cells were inoculated to 2 mL fresh RPMI-1640 media (or YPD for *S. cerevisiae* cells) that was supplemented with the designated inhibitor or DMSO (for control groups) in 15 mL culture tubes. The cells were incubated for three hours at 30 °C with shaking at 225 rpm. Propidium iodide (PI, Sigma) was added to the cell suspension at a final concentration of 10 µM. PI-treated cells were incubated for 30–60 min at 37 °C, harvested, resuspended in 1X phosphate buffered saline (PBS, 137 mM NaCl, 2.7 mM KCl, 8 mM Na_2_HPO_4_, and 2 mM KH_2_PO_4_, pH 7.0), and quantified using flow cytometry [15]. Data were collected from 10,000 cells using an Attune NxT Flow Cytometer (Life Technologies) equipped with a 50 mW, 488 nm LED laser and 530/30 nm emissions filters. Data were analyzed using Attune NxT Software v2.2. Experiments were repeated at least three times, and data that are presented are representative of one experiment.

### 2.5. Hyphal Assays

Yeast cells were grown for approximately 16–24 h at 30 °C with shaking at 225 rpm in YPD. The cells were diluted into 10% fetal bovine serum (FBS)/YPD medium or Spider medium (1% nutrient broth, 1% mannitol, 0.2% K_2_PO_4_, pH 7.2) supplemented with Mel56 or DMSO (for control groups) to a final concentration of approximately 1–3 × 10^7^ cells/mL in a final volume of 2 mL. Cell cultures were incubated at 37 °C with shaking at 225 rpm for two hours. Approximately 5 µL of cell suspension was spotted onto polylysine-coated slides and visualized at a total magnification of 1000× with an EVOS compound light microscope (Thermofisher, USA). The captured images were processed using ImageJ.

### 2.6. Mitochondrial Assays

Mitochondrial membrane potential (MMP) assays were performed as previously described [15]. Briefly, yeast cells were cultured and treated with Mel56 as described in the viability assays section. Following Mel56 treatment, the cells were washed with 1X PBS and incubated with the dye JC-10 (Sigma, USA) (final concentration of 10 µM) for one hour at 37 °C. JC-10 fluorescence was quantified by flow cytometry. The cells were treated with 10 µM of the mitochondrial membrane perturbant carbonyl cyanide 4-(trifluoromethoxy) phenyl hydrazone (FCCP; ABCAM, USA) as a positive control to profile cells with depolarized mitochondria. The experiments were repeated at least three times, and data that are presented are representative of one experiment.

## 3. Results and Discussion

### 3.1. Identification of C. albicans Chemical Inhibitors

**Figure 1:**
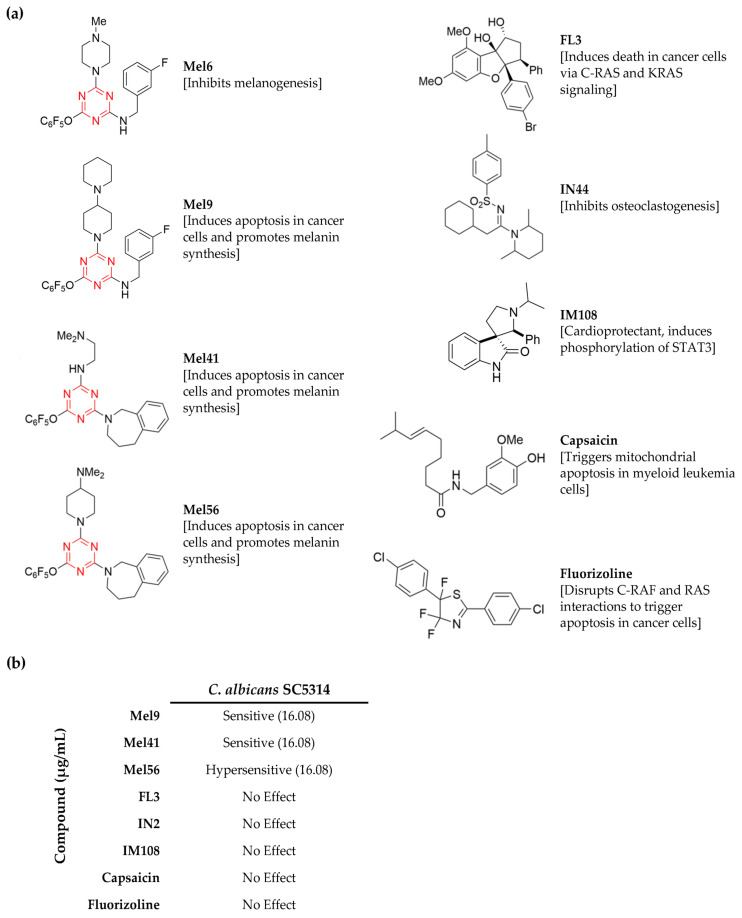
Analysis of prohibitin ligands that were used in this study. (**a**) Prohibitin ligands include the flavagline FL3 [30,31]; the melanogenin analogs Mel6, Mel9, Mel41, and Mel56 [17]; the sulfonylamidine IN44 [20]; the oxindole IM108 [21]; fluorizoline [28]; and capsaicin [19]. Pharmacological activities of the compounds are provided. For the melanogenin analogs, the triazine ring is colored in red. (**b**) *C. albicans* SC5314 cells were treated for 24 h at 30 °C with 5.35, 10.72, and 16.08 µg/mL of the designated inhibitor or DMSO (for controls). Growth was monitored in a microplate reader, and OD_600nm_ absorbance readings were recorded every 30 min. Candidate ligands that completely inhibited cell growth were chosen for subsequent MIC analysis. Growth phenotypes were assigned the following terms: hypersensitive (no growth observed in the microplate well), sensitive (growth was observed in the microplate well at approximately less than or equal to 50% of the mean OD_600nm_ value for the corresponding value of the untreated control group), and no effect (growth was similar to the DMSO-treated control groups).

A total of nine prohibitin inhibitor compounds from various chemical classes (Figure 1a) were screened in two types of growth assays using *C. albicans* strain SC5314. The first assay was designed to rapidly identify candidate compounds, and the second assay was performed to determine the MIC. The concentrations for each inhibitor tested were based on concentrations that were used to monitor apoptosis in human cancer cell lines (IC_50_ = 5.35 µg/mL) [17] and up to three times greater (10.72 and 16.08 µg/mL).

The compounds Mel9, Mel41, and Mel56 inhibited *C. albicans* growth, and the six remaining compounds had no effect (Figure 1b). The antifungal activity of Mel56 was the greatest; growth was not observed in wells containing Mel56 at a concentration of 16.08 µg/mL. On the other hand, 16.08 µg/mL of Mel9 and Mel41 was insufficient to completely inhibit *C. albicans* growth. Control samples that were grown in RPMI-1640 medium that was supplemented with DMSO showed no difference compared to samples that were grown in RPMI-1640 medium alone. Therefore, Mel56 was selected for further analyses. Similar results were obtained in growth assays using nutrient rich growth medium (YPD) or synthetic growth medium with varying carbon sources (YNB with glycerol or galactose) confirming that the Mel56 antifungal properties were independent of the growth medium.

### 3.2. Determination of Mel56 Minimum Inhibitory Concentration

To determine the MIC of Mel56 in *C. albicans*, broth double dilution assays were performed. As *C. albicans* frequently displays strain-specific phenotypes [32], Mel56 antifungal activity was monitored in *C. albicans* clinical isolates MC99 and MC102 and the laboratory reference strains SN250, DAY185, and DAY286. In addition, Mel56 antifungal activity was assessed in the fluconazole-resistant *C. albicans* clinical isolate 3147. Mel56 at a concentration of 8 µg/mL was comparable to MIC values that were obtained from samples that were treated with fluconazole (methods) and sufficient to completely inhibit the growth of *C. albicans* strains MC99, MC102, 3147, DAY185, DAY286, and SN250 compared to the DMSO-treated control groups following 24 h of growth (Table 1). The similarity of the MIC values for *C. albicans* strain 3147 compared to the other *C. albicans* strains suggests that the physiological basis for azole resistance in this strain is independent of Mel56 susceptibility.

**Figure 2:**
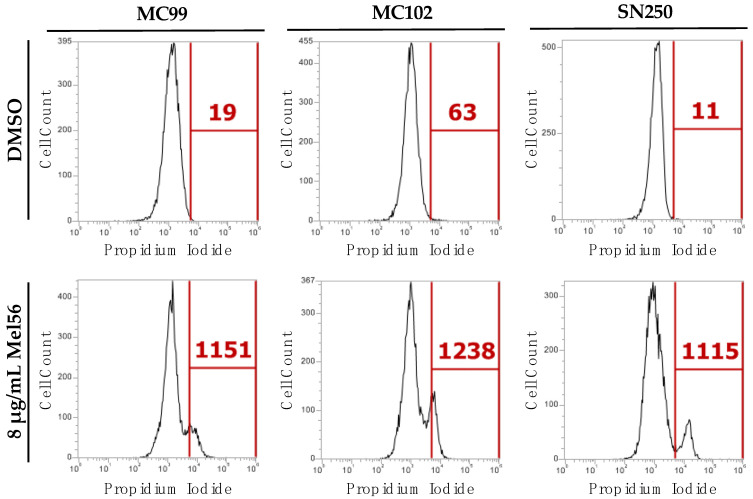
Viability analysis of *C. albicans* following prohibitin ligand treatment. Flow cytometry analysis of *C. albicans* strains SN250, MC99, and MC102 that were treated with 8 µg/mL Mel56 or DMSO (for controls) for three hours at 30 °C. Following incubation, the cells were harvested and labeled with PI. The number of PI-labeled cells are shown in the histograms (red font). Experiments were repeated three times and data presented is representative of one experiment.

To verify that Mel56 is fungicidal in *C. albicans*, yeast strains MC102, MC99, and SN250 were labeled with the viability dye PI. Labeling was initially performed by pooling samples directly from the microplate following 24 h of Mel56 exposure. However, PI labeling was poor due to extensive cell fragmentation and low cell recovery—both indicators of fungicidal activity. Therefore, we performed PI labeling following three hours of Mel56 treatment and with a more concentrated cell inoculum. In addition, these assays were performed using culture tubes and a shaker incubator. This design allowed us to assess O_2_ availability on Mel56 antifungal activity. In the microplate reader, the environment may be more anaerobic due to the volume capacity of the wells (250 µL well capacity containing a 200 µL cell suspension), microplate cover, and limited orbital shaking causing cells to settle at the bottom the well. There was a 20–100-fold increase in PI-positive cells after three hours of Mel56 treatment (Figure 2). Collectively, these findings demonstrate that Mel56 is fungicidal in *C. albicans* with a MIC of 8–16 µg/mL.

### 3.3. General Antifungal Activity of Prohibitin Ligands

The impact of Mel56 on *C. albicans* growth suggests that Mel56 may have general antifungal properties. Therefore, we examined Mel56 antifungal activity in several non-*albicans Candida* species and the yeast *S*. *cerevisiae*. MIC assay results for *C. tropicalis*, *C. parapsilosis*, *C. dubliniensis*, *C. glabrata*, and several *S. cerevisiae* strains show that Mel56 has general antifungal activity with MICs ranging from 4 to 8 µg/mL (Table 1). Notably, the MIC for Mel56 was lower in *C. glabrata* (4 µg/mL) compared to *C. albicans*. Among the non-*albicans Candida* species, *C. glabrata* remains one of the most treated fungal pathogens with azole-resistant species frequently isolated in nosocomial settings [33].

The triazine ring has been extensively used as a scaffold for a variety of synthetic compounds that target diverse biological targets. Accordingly, 1,3,5-triazine (s-triazine)-based compounds have demonstrated broad pharmacological activity including antimalarial [34], anticancer [35], antiviral [36], and antibacterial [37]. The low cytotoxicity and comparable MICs to common antifungals underscores the potential of triazine-derived compounds as antifungal agents [38,39,40]. Our findings with Mel56 expands the repertoire for this class of synthetic compounds. Mel56 is a derivative of Mel41 [17], and the weaker antifungal activity that was observed with Mel41 (Figure 1b) in *C. albicans* implies that the chemical basis for Mel56 antifungal activity resides in the 4-dimethylamino piperidine moiety which is absent in Mel41 (Figure 1a). Published data that associates 4-dimethylamino piperidine exclusively with antifungal activity is unavailable. However, a screen of 17 triazine compounds containing various moieties showed that compounds containing piperidine demonstrated the greatest antifungal activity in *C. albicans* [40]. Nevertheless, the successful applications of triazine-derivatives in a variety of microbial and cellular disease models [10] argues in favor for structural optimization experiments to address these issues.

### 3.4. Cellular Consequences of Mel56 Treatment

**Figure 3:**
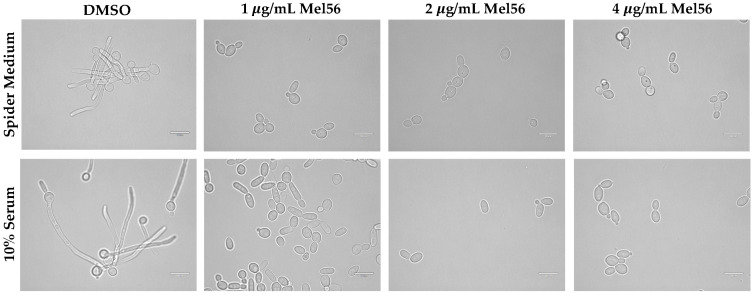
Inhibition of the yeast-hyphae transition by Mel56. *C. albicans* SC5314 cells were inoculated into 10% FBS/YPD or Spider medium that was supplemented with various concentrations of Mel56 (1, 2, and 4 µg/mL) or DMSO (for controls). Following two hours incubation at 37 °C, the samples were visualized at 1000× total magnification using bright-field light microscopy. The scale bars represent 10 µm.

The findings that are presented here (Figure 1 and Figure 2 and Table 1) and by other groups [8,38,39,40] clearly demonstrate the antifungal activity of triazine-derived compounds. However, the underlying cellular and molecular mechanisms remain elusive. Hyphal formation and growth are required for *C. albicans* pathogenesis, and hyphal-defective strains are avirulent [3]. Therefore, we examined the effect of acute Mel56 treatment at sublethal concentrations (1, 2, and 4 µg/mL) during the yeast-hyphae transition. Strikingly, bright-field light microscopy images revealed that Mel56 at concentrations ≥ 1 µg/mL suppressed *C. albicans* hyphal formation (Figure 3). Along with the critical role of hyphae in *C. albicans* pathogenesis, biofilm development is essential for commensalism and virulence and requires hyphal formation [41]. Thus, Mel56 can also be used to study biofilm dynamics. Taken together, these findings reveal a new role for triazine compounds as inhibitors of the yeast-hyphae transition. 

It must be noted that the hyphal assay environmental conditions (incubation temperature of 37 °C) are different compared to the broth dilution assay conditions (incubation temperature of 30 °C), whereby the latter promotes yeast cell growth exclusively. Although RPMI-1640 is used to induce hyphal growth, we did not observe hyphae in microplate assays for yeast cells that were grown in RPMI-1640 or RPMI-DMSO medium. Thus, it is plausible that the mechanisms of Mel56 antifungal activity and hyphal suppression are independent. Accordingly, dual roles of Mel56 have been observed in experiments using human cells. Mel56 treatment promotes both apoptosis in melanoma cells by inhibiting the AKT survival pathway and also promotes melanogenesis via upregulation of the melanogenic gene regulator, MITF. Both these activities require prohibitins [17]. Hyphal growth inhibition with sublethal concentrations of Mel56 provides an additional benefit to explore alternative therapeutic strategies with Mel56 such as combinatorial treatment [6]. Indeed, the results from a recent antifungal chemical screen by Xie et al. identified several triazine-derived compounds demonstrating synergistic activity with fluconazole, despite having no antifungal activity when used alone [8].

Melanogenin and melanogenin analogs bind to the human prohibitin proteins PHB1 and PHB2; however, the binding affinity and ligand binding site have not been determined [17]. Human and fungal prohibitins are associated with mitochondrial respiratory functions [42]. In *S. cerevisiae*, mitochondrial Phb1 and Phb2 assist in the assembly F_1_F_O_-ATP synthase complex by stabilizing the subunit Atp7 [43]. Therefore, we hypothesized that prohibitin ligand treatment disrupts mitochondrial respiratory function. Mitochondrial membrane potential (MMP) analysis was performed following prohibitin ligand treatment because disruption of MMP is indicative of electron transport chain dysfunction [44]. Also, in silico analysis of the primary amino acid sequence for *C. albicans* prohibitins predicts that these proteins localize to the mitochondrion [45]. *C. albicans* MC99, MC102, and SN250 yeast cells were treated with Mel56 or DMSO and labelled with the MMP fluorescent reporter probe JC-10. JC-10 is also used to monitor apoptosis-induced mitochondrial damage [15]; therefore, we treated *C. albicans* cells with acute Mel56 treatment (1-h) where PI labeling is not observed. JC-10-labelled yeast cells were analyzed with flow cytometry. We did not detect a significant change in MMP following acute Mel56 treatment compared to the DMSO-treated control groups (Figure S1). These findings suggest that Mel56 does not directly impair respiratory function.

### 3.5. Effect of Mel56 Treatment on C. albicans Prohibitin Mutants

To determine whether Mel56 directly disrupts prohibitin function in *C. albicans*, the growth of *C. albicans* prohibitin mutant strains was monitored in the presence of Mel56. If Mel56 targets prohibitins in *C. albicans*, then mutant strains should be resistant to Mel56 treatment. The *C. albicans* genome encodes three prohibitin genes: *PHB1*, *PHB2*, and *PHB12* [46]. CRISPR-Cas9 genome editing was employed to create a panel of *C. albicans* homozygous null mutant strains lacking *PHB1* or *PHB2* (single mutants), *PHB1* and *PHB2* (double mutant), and *PHB1*, *PHB2*, and *PHB12* (triple mutant).

Unexpectedly, the MIC assay results show that Mel56 antifungal activity in prohibitin mutant strains was similar to the wild-type parental strain SN250 (Table 1 and Table 2). These findings strongly imply that Mel56 may have a unique mechanism-of-action in *C. albicans* that is unrelated to SPFH proteins. In support of this idea, a 4,6-disubstituted *s*-triazine compound demonstrated antifungal activity against *C. albicans* by presumably targeting the enzyme *N*-myristoltransferase [40]. Also, the flavaglines class of prohibitin ligands targets both prohibitin and the initiation of translation protein, eIF4A to induce apoptosis in human cancer cell lines [10]. Whether flavagline-induced apoptotic activity is a consequence of attenuated prohibitin or eIFA function (or both) is unknown. Ultimately, genetic analysis, affinity-purification, and LC-MS/MS experiments will identify the Mel56 protein target in *C. albicans*.

## 4. Conclusions

We have tested the antifungal activity for a class of synthetic and natural compounds which target prohibitin proteins and were previously demonstrated to have therapeutic potential for various mammalian diseases. We found that the 1,3,5, triazine-based synthetic compound Mel56 exhibited antifungal activity against the major human fungal pathogen *C. albicans*, several non-*albicans Candida* strains, and *S. cerevisiae*. However, the cellular and molecular mechanisms underlying Mel56 antifungal activity remain unresolved. Our findings strongly support a model that Mel56-based inhibition is independent of prohibitin proteins in *C. albicans*, but we cannot completely rule out the possibility that Mel56 targets other SPFH proteins.

Remarkably, Mel56 inhibits the yeast-to-hyphae transition at concentrations significantly lower than the observed MICs. These findings can help guide the optimization of Mel56 as a fungicidal or fungistatic compound or both. In-vivo infection assays will inform on the efficacy of both strategies. For example, the invertebrate planarian *Schmidtea mediterranea* is an excellent host to study the impact of Mel56 treatment in *C. albicans* pathogenesis. The simple planarian anatomical design allows visualization of all phases of *C. albicans* infection, such as adherence, yeast-hyphae transition, and invasive growth which cannot be observed in vertebrate infection assays [47]. Thus, triazine-based compounds such as Mel56 can pave the way in bolstering the antifungal arsenal.

## Figures and Tables

**Table 1:** MIC determination of Mel56 in various fungal species.

Compound MIC [µg/mL]	
Yeast Strain	Mel56
*C. albicans* SC5314	16
*C. albicans* SN250	8
*C. albicans* DAY185	8
*C. albicans* DAY 286	8
*C. albicans* MC99	8
*C. albicans* MC102	8
*C. albicans* 3147	8
*C. glabrata*	4
*C. tropicalis*	8
*C. parapsilosis*	8
*C. dubliniensis*	8
*S. cerevisiae* BY4741	4
*S. cerevisiae* BY4742	4
*S. cerevisiae* W303-1A	4
*S. cerevisiae* W303-1B	4

**Table 2:** MIC determination of Mel56 in *C. albicans* prohibitin mutant strains.

Compound MIC [µg/mL]	
Yeast Strain	Mel56
*C*. *albicans phb1Δ/Δ*	8
*C*. *albicans phb2Δ/Δ*	8
*C*. *albicans phb1Δ/Δ-phb2Δ/Δ*	8
*C*. *albicans phb1Δ/Δ-phb2Δ/Δ-phb12Δ/Δ*	8

## Data Availability

Not applicable.

## Supplementary Materials

The following supporting information can be downloaded at: https://www.mdpi.com/article/10.3390/pathogens12010126/s1, Figure S1: Mitochondrial analysis of *C. albicans* following Mel56 treatment; Table S1: Oligonucleotides that were used in this study.
