# Supplementary figures and images for: Triazine-Based Small Molecules: A Potential New Class of Compounds in the Antifungal Toolbox
Source: Pathogens. 2023 Jan 12;12(1):126. doi: 10.3390/pathogens12010126 (PMC9861074; doi:10.3390/pathogens12010126)

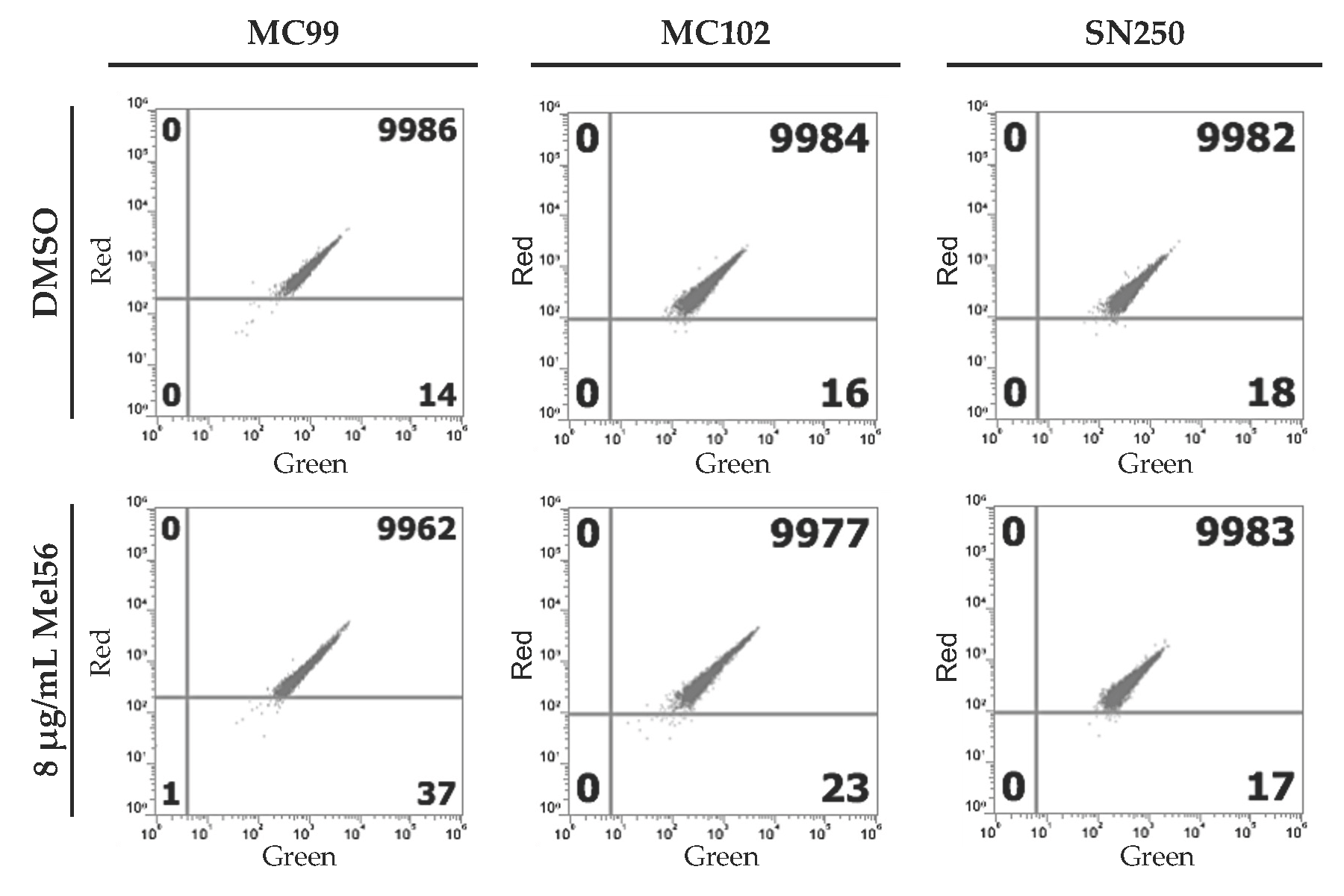

Supplement: Supplementary file 1 [file pathogens-12-00126-s001.zip › pathogens-2149407-Figure S1.tiff]
